# Temporal changes in physical fitness in Norwegian male and female military conscripts between 2006 and 2020

**DOI:** 10.1111/sms.14238

**Published:** 2022-11-04

**Authors:** Anders Aandstad

**Affiliations:** ^1^ Section for Military Leadership and Sport Norwegian Defence University College Oslo Norway

**Keywords:** endurance, muscle strength, recruits, secular, soldiers

## Abstract

Reduced physical fitness has been documented in Western children and adults over the past five decades. The same trend has been observed among soldiers, but the number of studies is scarce. Thus, the aim of the present study was to investigate temporal changes in physical fitness in Norwegian conscripts. All conscripts who performed entry fitness tests between 2006 and 2020 were included in the study (*n* = 105 100; 17% females). Endurance was measured with the 3000 m run, while push‐ups, sit‐ups, and pull‐ups (2006–2016) and medicine ball throw, standing long jump, and pull‐ups (2017–2020) were used to measure muscle strength. Mean (95% confidence intervals) 3000 m run time was reduced by 52 (47, 57) seconds in men, 90 (76, 105) seconds in women, and 16 (11, 20) seconds in both sexes combined. Muscle strength increased statistically significantly in four out of five tests in men, three out of six tests in women, and two out of five tests for both sexes combined. Effect sizes for statistically significant changes ranged from 0.06 to 0.82. In conclusion, Norwegian conscripts improved their cardiorespiratory endurance between 2006 and 2020, with improvements observed for most muscle strength tests. When analyzing both sexes combined, the improvements diminished. The latter is attributed to a sevenfold increase in relative number of female conscripts over the 15‐year period. The present findings should not be generalized to all young Norwegian men and women since conscripts are selected based on fitness, and only ~15% of the population end up serving.

## 1 INTRODUCTION

Over the past five decades, reduced cardiorespiratory endurance levels have been reported among children and adults in the Western world, albeit some data indicate that the decline has diminished or plateaued since the new millennium.^1^, ^2^, ^3^ Temporal changes in muscle strength seem less clear and may depend on factors such as type of strength and whether the test included moving external load or the subjects' body weight.^2^, ^4^, ^5^

Norwegian population studies on trends in cardiorespiratory endurance are consistent with the international data, indicating declines in adolescents and young adults.^6^, ^7^, ^8^ Less information is available pertaining temporal changes in muscle strength among Norwegians, with existing studies presenting equivocal conclusions.^7^, ^9^

Physical fitness may be defined and categorized in several ways, but cardiorespiratory endurance, muscle strength, and muscular endurance are typically considered key elements of (health‐related) physical fitness.^10^ In a military context, higher levels of fitness are linked to better job performance, better health, less injuries, and lower attrition rates.^11^, ^12^, ^13^ The military therefore strive to optimize soldier's physical fitness through appropriate selection and physical training.

Military personnel are recruited from the civilian population, and temporal trends in civilian's physical fitness may therefore be transferred to the military community. However, armies usually select their soldiers based on certain physical fitness standards, and this will affect physical fitness levels of serving personnel. Some countries practice obligatory military conscription for nearly all young men (and women), while other countries rely on voluntary professional soldiers. Such differences may also impact soldiers' physical fitness levels. Thus, temporal trends in an army's fitness level may not follow the country's civilian trend or trends in other nations' military forces. Military‐specific studies are therefore needed and should be performed on regular basis.

Yet relatively few studies have investigated temporal trends in physical fitness for soldiers. Reduced performance in distance or time‐based running tests have been reported in male and/or female recruits or soldiers from the US Army,^14^, ^15^, ^16^ Finland,^17^, ^18^, ^19^ and Poland.^20^ At the same time, little or no changes in muscle strength and muscular endurance have been reported among US Army and Finnish recruits and soldiers.^14^, ^15^, ^16^, ^17^, ^19^ Similar studies have not yet been conducted on Norwegian soldiers.

The Norwegian conscription model has changed over time. During the last 15 years, only ~15% of all young men and women were required to conduct the 1‐year long conscript service,^21^, ^22^ which is considerably less than during the 1980–1990s. Moreover, far more women serve today than a decade ago. This is primarily caused by the authorities' decision to make conscript selection and service obligatory also for women.^22^ Together with the re‐introduction of physical fitness tests at conscript selection in 2011,^23^ these structural changes may have affected the physical fitness level of Norwegian conscripts.

Systematic fitness testing of serving Norwegian conscripts was introduced in the early 1970s. However, electronically saved individual data were only available from the mid‐2000s. Accordingly, the aim of the present study was to investigate changes in cardiorespiratory endurance and muscle strength in Norwegian conscript soldiers from 2006 to 2020.

## 2 MATERIAL AND METHODS

This study can be characterized as an observational pseudo‐longitudinal (repeated cross‐sectional) study. It was approved by the Research Group at the Norwegian Defense University College, while the Norwegian Centre for Research Data and the Regional Committee for Medical and Health Research Ethics considered the study to be exempted from notification due to use of anonymous register data only. The data were extracted from the P3 database by technical personnel from the Norwegian Armed Forces HR and Conscription Centre (Hamar, Norway).

### 2.1 Subjects

**FIGURE 1:**
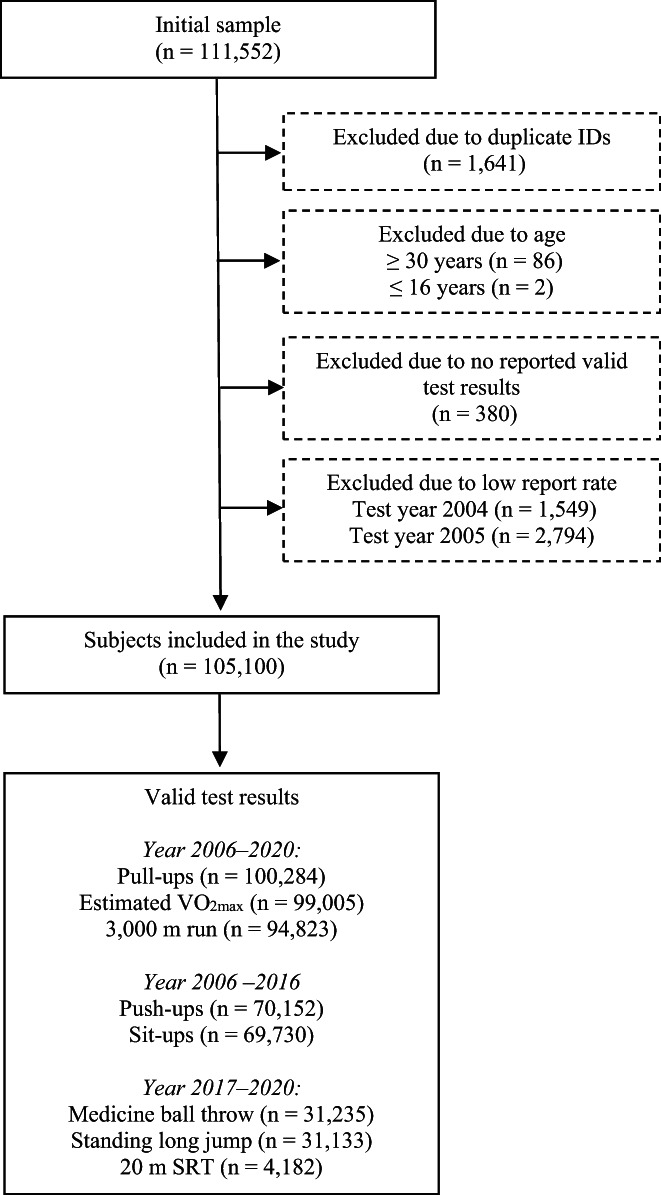
Flowchart illustrating the number of subjects excluded and included in the study, as well as the number of valid tests included in the analyses

All Norwegian male and female military conscripts (recruits) registered in the P3 database with physical fitness entry test results up until year 2020 were first identified (*n* = 111 552). Data from the two first years (2004 and 2005) were excluded due to low participation rates (~15% and ~35%, respectively). Other exclusion criteria are presented in Figure 1.

Accordingly, 105 100 conscripts were included in the study, consisting of 87 573 men (83%) and 17 527 women (17%). All had produced at least one valid test result between year 2006 and 2020. Participation rate was approximately 80%–85%, based on previous publications reporting annual number of serving conscripts.^22^, ^24^ Age ranged from 17 to 29 years, and mean (SD) age of men and women were 19.4 (1.2) and 19.1 (1.0) years, respectively. The included subjects served in the Army (60%), Navy (21%), or Air Force (19%).

The number of reported results vary among the different test variables (Figure 1). This is because some conscripts could not perform all tests due to injuries, sickness, or because they were absent during testing (conflicting military duties, etc.). Moreover, some results were excluded as they were considered mistyped in the database (see statistical analyses). Finally, two muscle strength tests were replaced by two new tests in 2017, which led to lower sample size for the four involved muscle strength tests.

### 2.2 Measurements

Testing was administered within the first 3 weeks after entry to conscript service and took place at multiple military bases and at different times of the year. All tests were completed on one or separate days, in parallel to their regular military service. Sports officers and their conscript assistants acted as test leaders. The conscript soldiers performed the tests dressed in running shoes and sports attire. A minimum of 15 min were allocated to warm‐up exercises prior to testing. The tests were performed according to the Norwegian Armed Forces official test regulations.

### 2.3 Cardiorespiratory endurance

#### 2.3.1 3000 m run

Testing took place outside on a flat course (tarmac, firm gravel, or snow covered by sand). Acceptable temperature limits were set at −15 to +25°C. Running time was registered by manual stopwatches, or by automatic timing chips. The conscripts started the test in groups of varying sizes and were encouraged to finish the distance as fast as possible. Performance was registered as run time in minutes and seconds, which was later converted to seconds, minutes, and average run speed (km∙h^−1^) for the analyses. The 3000 m run acted as the only (2006–2016) or primary (2017–2020) cardiorespiratory endurance test over the time period. A recent validation study in Norwegian military personnel reported Pearson *r* between directly measured VO_2max_ and average 3000 m run speed to be 0.74 and 0.79 in men and women, respectively.^25^ Sex‐specific prediction equations (Supporting Information S1) from this validation study were used to convert run performance into estimated VO_2max_.

#### 2.3.2 20 m shuttle run test (20 m SRT)

This test was available for the years 2017–2020 as an indoor alternative to the 3000 m run. The subjects ran back and forth (in groups) between two lines 20 m apart, while running speed was dictated from audio bleeps.^26^ Initial speed was 8.0 km h^−1^ at level 1, 9.0 km h^−1^ at level 2, and thereafter increased by 0.5 km·h^−1^ at every new level. The test ended when the subject stopped running because of fatigue or when he or she was unable to reach the line on three consecutive occasions (≥3 m from the line). The result was registered as number of levels and shuttles completed but converted to total number of shuttles and last half‐level completed for the analyses. Validity of the 20 m SRT is previously reported in several studies on adults,^26^ including male Norwegian military personnel.^27^ These studies have typically reported moderate to high correlations (Pearson *r* = 0.65–0.90) between 20 m SRT performance and directly measured VO_2max_. In the current study, sex‐specific prediction equations developed by Stickland et al^28^ (Supporting Information S1) were used to calculate estimated VO_2max_ based on run performance.

### 2.4 Muscle strength

Between year 2006 and 2016, muscle strength was measured by a test battery consisting of push‐ups, sit‐ups, and pull‐ups. Execution and validity of these tests have been presented earlier,^29^ but a brief explanation is also given below. These tests were replaced by medicine ball throw, standing long jump and a sex‐neutral pull‐up test from 2017 and onwards.

#### 2.4.1 Push‐ups

The test was performed from a starting position on the floor, with hands shoulder‐width apart and with a straight body. The subject then raised his or her body until the arms were fully stretched, followed by lowering the body until the chest touched the test leader's flat hand held on the floor. The test was performed continuously, and with no time limitation. Results were registered as the number of accepted repetitions.

#### 2.4.2 Sit‐ups

The subject started from a supine position on a mat, with legs placed at the upper section of a vaulting box and with the knees bent 90 degrees. The test leader fixed the legs by pressing the ankles to the box. The subject then raised his or her upper body so that the elbow touched the opposite knee. Every other repetition was performed to the right and left knee, respectively. The test was performed continuously, and with no time limitation. Results were registered as the number of accepted repetitions.

#### 2.4.3 Pull‐ups

For the years 2006–2016, the test was administered vertically for men and horizontally for women (illustrations presented in a previous publication).^23^ The starting position for men was hanging vertically from a beam using an overhand grasp and with straight arms and legs. The subject then raised his body until the chin was over the bar, followed by lowering the body until the arms were fully stretched. This cycle was repeated until fatigue. The starting position for women was hanging horizontally from a beam using an overhand grasp and with straight arms and legs. The heels were placed on a bench to achieve a horizontal starting position. The subject then raised her body until the chest touched the beam (repeated until fatigue). For the years 2017–2020, both men and women first performed the vertical version of the pull‐up test. If the subject was not able to perform any vertical pull‐ups, the horizontal version of the pull‐up test was administered. The number of accepted repetitions (vertical or horizontal) was registered.

#### 2.4.4 Medicine ball throw

This test was performed from an upright starting position with the feet placed shoulder‐width apart behind a line.^29^ A 10‐kg rubber medicine ball was held against the chest. The subject then flexed his or her ankles, knees, and hips and forcefully extended his or her arms to propel the ball forward. The feet were not allowed to lose contact with the floor or touch the line on the floor during the throw. The length of the throw was measured from localizing the center of the ball's impact point to the nearest 10 cm by use of a customized measurement mat or a tape measure. Three trials were performed, and the best result was recorded.

#### 2.4.5 Standing long jump

The standing long jump was performed with the subject standing behind a line on a mat or the floor.^23^ The ankles and knees were flexed, and arms were swung to enhance the forward propelling movement of the body in an attempt to jump as far as possible. The landing spot for the most rear part of the shoes (or body) was identified, and the jump was measured to the nearest 5 cm. It was not necessary to stand still after landing on the mat. The best result of three attempts was recorded.

### 2.5 Statistical analyses

All outcome variables were checked for normality by visual inspections of histograms. Pull‐ups were considered skewed, as well as push‐ups for women. However, due to the large sample size, parametric statistical methods were used when analyzing all variables.^30^

The original data set was inspected for mistyped data points in the P3 register. A description of the data cleansing process is given in Supporting Information S2. For any test variable, the percentage of excluded or corrected data points was ≤0.42% of the total valid sample size.

All data were analyzed and presented for each sex separately, and for the two sexes combined. Descriptive data are presented as means with standard deviation (SD) or 95% confidence intervals (CI).

Linear regression was used to analyze the trend in physical fitness over time. The test variable was set as dependent variable, while test year represented the independent variable. The unstandardized beta coefficients (*B*) are presented, including their 95% CI where applicable.

Change between the first and the last year of testing was analyzed by an independent sample *t* test and presented as mean (95% CI) and percent change. Effect sizes (ES) calculated as Cohen's *d* supplemented these analyses. When applicable, the ES were interpreted as trivial (≤0.19), small (0.20–0.49), medium (0.50–0.79), and large (≥0.80).^31^ Cumulative relative frequency and percentile values (distribution of scores) for the first and last year of testing for all variables were also calculated and presented.

Statistical analyses were performed in SPSS (version 28, IBM Corp., Armonk, New York, USA) and Graphpad Prism (version 9.3.0, Graphpad Software, La Jolla, CA, USA). A *p*‐value of <0.05 was considered statistically significant.

## 3 RESULTS

**FIGURE 2:**
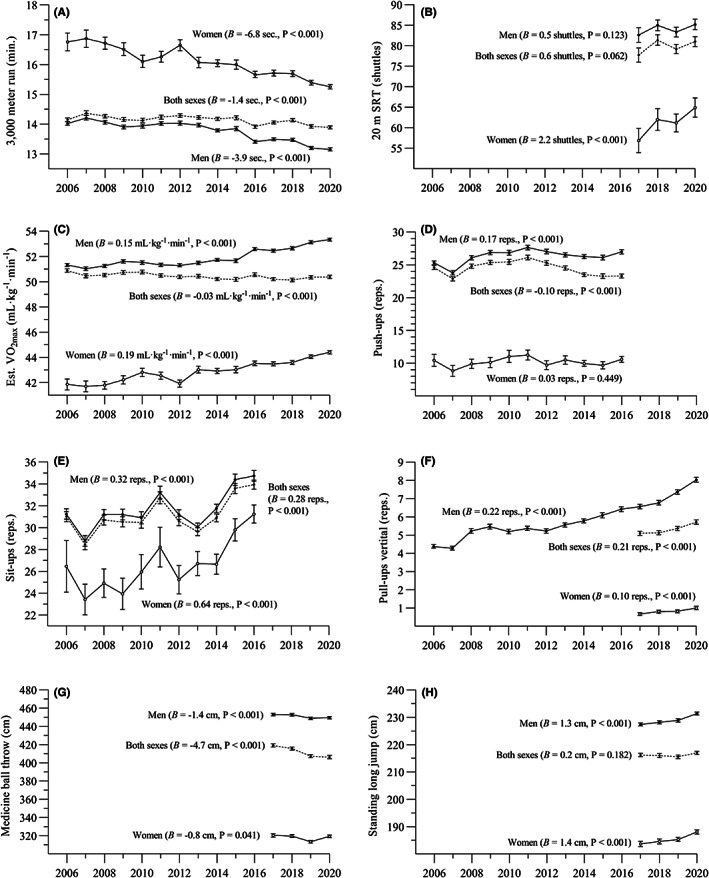
Temporal changes in physical fitness in Norwegian conscripts (● men; **○** women; ♦ both sexes combined) between 2006 and 2020. The panels represent (A) 3000 m run; (B) 20 m shuttle run test, (C) estimated maximal oxygen uptake, (D) push‐ups, (E) sit‐ups, (F) vertical pull‐ups, (G) medicine ball throw and (H) standing long jump. *B* = unstandardized beta coefficient (annual change)

**FIGURE 3:**
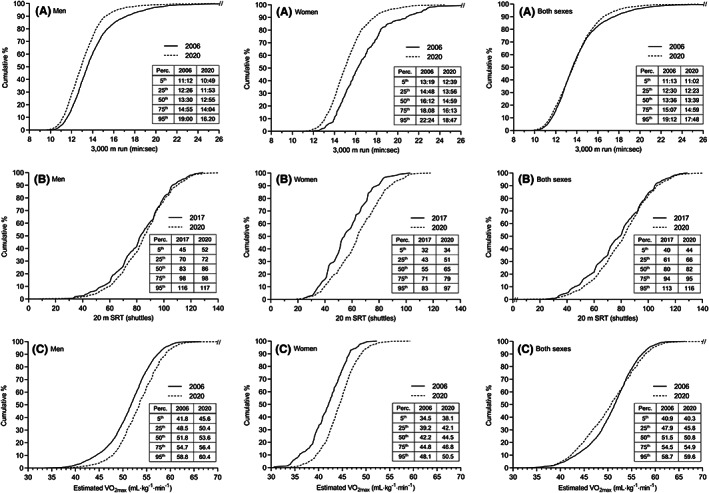
Cumulative relative frequency and selected percentile (perc.) values for cardiorespiratory endurance variables. The panels represent (A) 3000 m run, (B) 20 m shuttle run test and (C) estimated maximal oxygen uptake. Data are presented for men, women and both sexes, respectively

Cardiorespiratory endurance improved statistically significantly in both male and female recruits from 2006 to 2020 (see Figure 2a–c and Supporting Information S3–S5). Over this period, average 3000 m run time was reduced by 52 (47, 57) seconds (6.2%, ES = 0.39) in men, and 90 (76, 105) seconds (8.9%, ES = 0.74) in women. Also 20 m SRT performance increased in men (3.1%, ES = 0.13, P = 0.019) and women (14.2%, ES = 0.45, *p* < 0.001) between 2017 and 2020. Based on performance in the two running tests, mean estimated VO_2max_ increased by 2.0 (1.8, 2.2) ml kg^−1^ min^−1^ (3.9%, ES = 0.42) in men and 2.5 (2.1, 3.0) ml kg^−1^ min^−1^ (6.0%, ES = 0.68) in women between 2006 and 2020. When analyzing cardiorespiratory endurance in both sexes combined, trivial temporal improvements were observed for the 3000 m run (1.9%, ES = 0.11, *p* < 0.001) and the 20 m SRT (4.2%, ES = 0.15, *p* = 0.002), while estimated VO_2max_ were slightly reduced over the 15‐year period (1.0%, ES = 0.09, *p* < 0.001). In men and women analyzed separately, there was also a positive change in percentile distributions over the years, i.e., relatively fewer men and women with low endurance levels and more with high levels (Figure 3). In men and women combined, only minor temporal changes in percentile distributions were observed.

**FIGURE 4:**
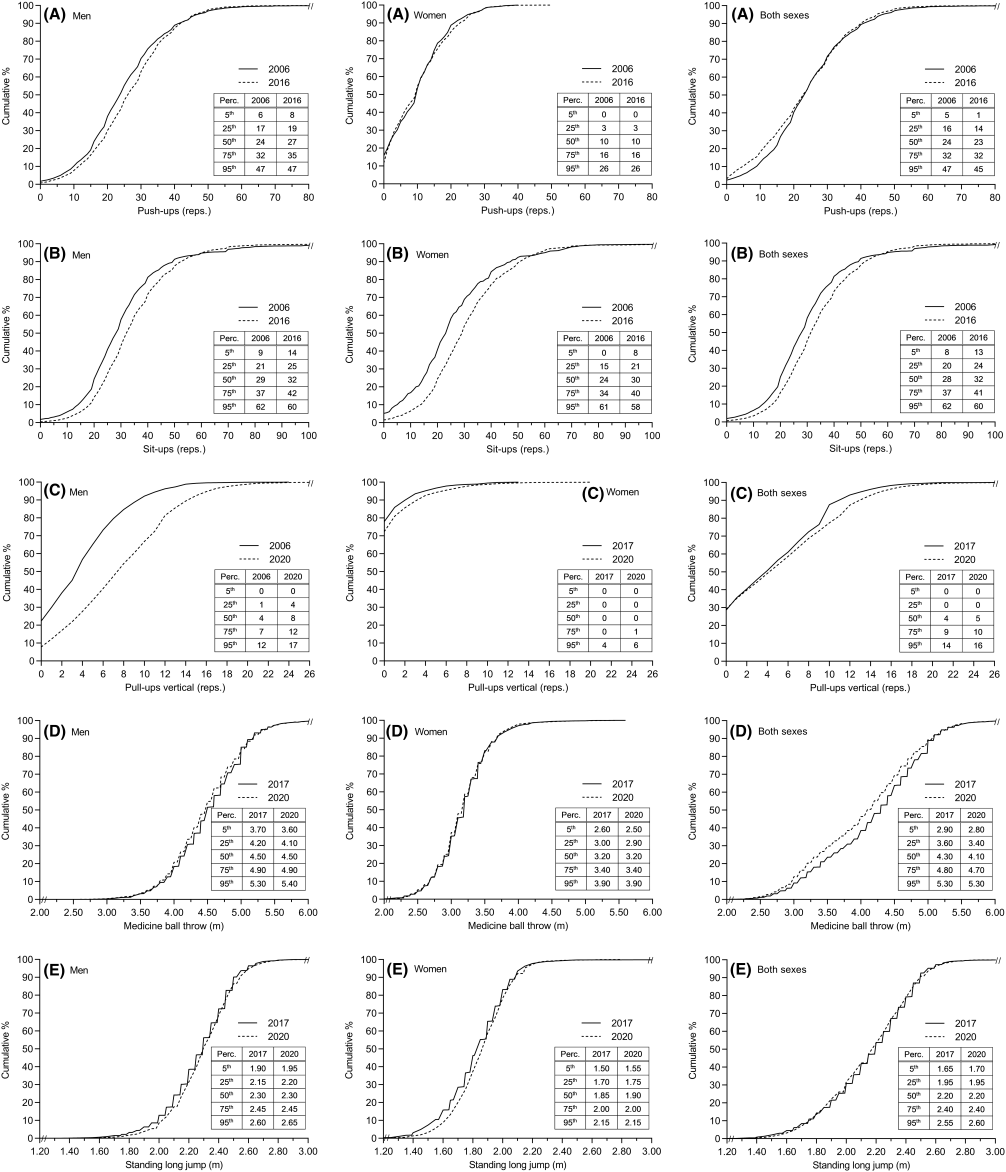
Cumulative relative frequency and selected percentile (perc.) values for muscle strength variables. The panels represent (A) push‐ups, (B) sit‐ups, (C) vertical pull‐ups, (D) medicine ball throw and (E) standing long jump. Data are presented for men, women and both sexes, respectively

Temporal changes in muscle strength are presented in Figure 2d–h and in Supporting Information S6–S11. In men, statistically significant improvements were observed in four out of five muscle strength tests. Vertical pull‐ups improved by 84% (ES = 0.82, *p* < 0.001), while push‐ups, sit‐ups and standing long jump increased by 2%–11% (ES = 0.14–0.19, all *p* < 0.001). Contrary, performance in medicine ball throw was reduced by 0.7% (ES = 0.06, *p* < 0.001). In women, improvements were observed for sit‐ups (18%, ES = 0.27, *p* < 0.001), vertical pull‐ups (43%, ES = 0.16, *p* < 0.001), and standing long jump (2.2%, ES = 0.22, *p* < 0.001). No change was observed for push‐ups (*p* = 0.790) and medicine ball throw (*p* = 0.411), while horizontal pull‐ups performance was reduced by 11% (ES = 0.12, *p* = 0.040). When analyzing both sexes combined, temporal improvements in performance were observed for sit‐ups (9.4%, ES = 0.16, *p* < 0.001) and vertical pull‐ups (12%, ES = 0.12, *p* < 0.001), while a reduction was observed for push‐ups (5.3%, ES = 0.10, *p* < 0.001) and medicine ball throw (3.1%, ES = 0.16, *p* < 0.001). Standing long jump performance was unchanged for both sexes combined (*p* = 0.086). Temporal changes in percentile distributions for muscle strength showed a positive development in men (i.e., relatively fewer subjects with low muscle strength levels, and more with high levels), with medicine ball throw as an exception (Figure 4). Similar analyses for women and both sexes combined were less clear, as results varied among the different muscle strength variables.

The number of women included in the analyses (n and percent of total sample) increased markedly over the years (see Supporting Information S3–S11). As an example, while women constituted 5% of the reported 3000 m run results in 2006, this number was 35% in 2020. Additionally, men produced statistically significantly higher mean scores compared to women for all test variables at all time points (Figure 2a–h).

## 4 DISCUSSION

This study investigated temporal changes in physical fitness in Norwegian male and female conscripts between 2006 and 2020. Statistically significant improvements in cardiorespiratory endurance were observed in both men and women. Muscle strength, power and muscular endurance also demonstrated improvements in four out of five tests for men, and three out of six tests for women. All improvements diminished considerably when male and female soldiers were analyzed together, which is attributed to the sevenfold increase in women tested in 2020 compared to 2006.

Temporal trends in physical fitness in recruits have been most comprehensively reported for Finland,^17^, ^19^ and the US Army.^15^, ^16^ These studies demonstrated reductions in distance run performance among male and female soldiers at entry to military service, and generally no or small changes in muscle strength, power, and muscular endurance. The positive development in physical fitness among Norwegian male and female conscripts is therefore in conflict with earlier findings, particularly for cardiorespiratory endurance. There are several possible reasons for this discrepancy. Firstly, the present study covered the years from 2006 to 2020, while the Finnish and American studies investigated secular changes over a longer period, starting from the 1970–1980s. There are indications that most of the decline in physical fitness among civilians and military personnel occurred before year 2000, and that changes have plateaued after that.^2^, ^17^, ^32^ Thus, if the present study had covered a longer period the conclusions may have been altered.

The plateau or more modest decrements in fitness observed in other countries' military forces after year 2000 is still in conflict with the rather strong positive development among Norwegian conscripts in the same period. Thus, a second reason for the observed discrepancy is likely related to recent changes in the Norwegian conscription system, and differences in this system compared to other countries. Norway introduced compulsory gender‐neutral conscript selection and service in 2010 and 2016, respectively.^22^ This has increased the number of female conscripts markedly and simultaneously made it easier to select women with higher fitness levels. Before introducing compulsory service for women, the relatively few women who volunteered were typically offered service as long as some rather low physical fitness standards were fulfilled. Today, the military chooses among all women (and men) from the entire population, and high‐fit subjects are usually preferred during selection. At the same time, the number of serving conscripts has been relatively stable between year 2006 and 2020 (8–9000 annually),^21^, ^22^ which constitutes only ~15% of all young Norwegian men and women. This means that less men serve today than 15 years ago, which makes it possible to also select harder on fitness among male conscripts. The current Norwegian conscription model is therefore fundamentally different to countries like Finland and Switzerland, which practice obligatory conscription only for men, and where the majority of men need to serve.^17^, ^32^ The Norwegian conscription system is also very different from the volunteer system used by for instance the US Army.

Another reason for the observed positive trend could be attributed to the introduction of a two‐step conscript selection system in 2010. In step one, all men and women aged 17 years old are required to report their fitness level in an online questionnaire. In step two (a year later), the pre‐selected subjects perform a maximal treadmill test and muscle strength tests at a recruitment center.^23^ Subjects with higher fitness levels are more likely to end up serving as conscripts. No objective tests were administered at conscript selection between the mid‐1990s and 2010. Thus, the increase in fitness among Norwegian conscripts between 2006 and 2020 may partly be credited to improvements in the selection system.

Finally, it should be mentioned that recent changes in the system for recruiting officers to the Norwegian Armed Forces may potentially have influenced the last years' results. Since ~2018, subjects who seek a career as non‐commissioned officer first need to serve as a conscript and are therefore included in the present 2018–2020 data. Yet, while fitness level is likely somewhat higher in the included prospective non‐commissioned officers,^33^ only ~2–300 subjects a year serve as conscripts prior to conducting non‐commissioned officers education. Accordingly, this factor will only marginally influence mean fitness values for recent years and has not impacted the trend prior to ~2018.

The present study only covered changes in physical fitness over the last 15 years. Yet, earlier cross‐sectional data of Norwegian conscripts may be used to get some insights into a more long‐term development. As early as 1923, physical fitness was evaluated from seven field tests in 3600 male military recruits at different bases in Norway.^34^ Pull‐up was the only test that remains today and can be used to analyze secular changes. The mean (SD) number of pull‐ups almost 100 years ago was 8.8 (3.3) repetitions, compared to 8.0 (5.1) in male conscripts in 2020. Thus, while mean pull‐up performance was clearly lower 15 years ago, it is now almost back to 1923 level. Yet, testing at that time was not described in detail, and it is possible that pull‐up testing was performed slightly different than today.

Between 1981 and 2002, the Norwegian Armed Forces published annual reports of 3000 m run performance in all conscripts tested at entry.^35^, ^36^ These documents only reported the percentage of conscripts who fulfilled the set minimum requirements (15:00 and 16:30 min:sec in men and women, respectively). The reports from the three first years were unavailable. Data from remaining reports had to be standardized due to differing tabulations. Accordingly, from 1984 to 2002 the annual passing rate for the 3000 m run ranged from 72% to 79% (76% in average). Similar analyses of the current data demonstrate that 86% of the 2020 conscripts reached the same 3000 m run standards (data not shown). Apparently, fewer conscripts have low cardiorespiratory endurance today compared to two to four decades ago. However, other methodological factors such as the increase in serving women and differences in annual participation rate may influence the results and hamper direct comparisons.

The present study's findings should be encouraging for the Norwegian Armed Forces, as physical fitness has increased in both male and female conscripts since 2006. Although seven times more women serve as conscripts today compared to 15 years ago, the mean physical fitness levels (irrespective of sex) are overall maintained. Moreover, cardiorespiratory endurance of Norwegian conscripts compares favorably to young male and female recruits from other countries. The average 3000 m run speed for Norwegian male conscripts in 2020 was 13% faster than for Finnish males tested in 2015 at the 12‐min run (13.7 and 12.1 km h^−1^, respectively).^17^ Corresponding analyses for Finnish and Norwegian female conscripts demonstrate 8% difference.^19^ Similarly sized differences in mean running speed are also found between Norwegian conscripts and US Army recruits performing the 2‐mile run (3.2 km).^37^ For muscle strength, power and muscular endurance, Norwegian conscripts generally demonstrate fewer repetitions in push‐ups and sit‐ups compared to Finnish and US Army recruits, but better standing long jump performance.^17^, ^19^, ^37^ However, differences in test executions may complicate such direct comparisons.

In military units it is important that all soldiers are above certain minimum levels for physical fitness (“a chain is no stronger than its weakest link”). Beside changes in mean values, it is therefore also interesting to study changes in distributions, especially the relative number of subjects with low fitness. Overall, the present study found parallel temporal changes in mean values and distributions. For instance, the observed improvements in mean 3000 m run time in both men and women were accompanied by a reduced number of male and female conscripts with slow run times (and more with fast times). Thus, from an occupational perspective, the observed positive temporal trend of fewer low‐fit male and female conscripts is worth emphasizing.

Despite the positive development pertaining physical fitness among Norwegian conscripts there are still room for improvement. Earlier task and demand analyses of military work (loaded marching, etc.) have indicated that a VO_2max_ of 43–50 ml kg^−1^ min^−1^ will be necessary to complete such tasks safe and efficiently.^38^, ^39^ Among all Norwegian conscripts tested in 2020, 13% possessed a VO_2max_ of less than 43 ml kg^−1^ min^−1^ (34% of all women, 2% of men), while 47% were below the 50 ml kg^−1^ min^−1^ threshold (95% of women, 21% of men). Moreover, 29% of the conscripts were not able to perform one vertical pull‐up repetition (72% of women, 8% of men). Based on previous data from selection step two there are certainly enough prospective conscripts who would fulfill the abovementioned criteria.^23^ Thus, it is possible to implement more strict minimum requirements for physical fitness, and still recruit 8–9000 subjects a year to conscript service. Yet, the consequences would likely be fewer serving women, and less emphasis on other important selection criteria, such as medical health, education, and psychological factors. Thus, the military should frequently evaluate their selection criteria and strive to optimize the balance between soldiers' physical work capacity and other operational requirements.

### 4.1 Study strengths and weaknesses

The present study included a large sample size, as all conscripts who performed entry tests over the last 15 years were included in the analyses. Earlier military research has often included male participants only, while the present study included both sexes. Objective maximal tests were used to investigate cardiorespiratory endurance and muscle strength, power, and muscular endurance. Since motivation for service was a selection criterion for the soldiers, it is assumed that most of the subjects were motivated to give their best effort—which is a prerequisite for high validity of maximal performance tests.

There are also some potential weaknesses associated with the present study. Firstly, physical fitness data is missing from ~15% to 20% of the conscripts. Reasons for individuals' missing data are unknown but probably attributed to early attrition (discharged within the first 3 weeks after entry), sickness, injuries, or being absent for other reasons. We may assume that fitness level is somewhat lower in subjects not being tested, but no data are available to verify this. Yet, participation rates (80%–85%) have been stable over the 15‐year period and the missing data will therefore likely have little or no impact on the temporal trend analyses. Another methodological disadvantage is related to changes in the test regulations from year 2017. The introduction of new tests in 2017 particularly complicates temporal trend analyses of muscle strength. Thus, when comparing percent changes and effect sizes among different tests, the reader should have in mind that the figures may represent 4‐, 11‐, or 15‐year changes. The test score tables also changed in 2017, which may have impacted conscripts' motivation and effort during testing. Other potential weaknesses include possible mistyping of test data in the registry (only obvious mistakes were excluded), possible differences in how test leaders administered the tests at the different military bases, and the lack of height, weight, and BMI data—which could have helped in explaining observed fitness trends.

Finally, it must be emphasized that today's Norwegian conscripts does not represent the general population as they are selected based on physical fitness and because only ~15% of the population serve as conscripts. Accordingly, the present findings should not be used to make conclusions regarding temporal trends in physical fitness in the general Norwegian population.

## 5 PERSPECTIVE

Male and female Norwegian conscripts have increased their cardiorespiratory endurance between 2006 and 2020. Muscle strength, power and muscular endurance also increased in four out of five tests in men, and three out of six tests in women. In particular, the clear increase in cardiorespiratory endurance contradicts earlier findings in soldiers from other Western countries.^14^, ^17^

The relative number of women serving as conscripts in the Norwegian Armed Forces has increased sevenfold during the last 15 years. Although women generally possess lower cardiorespiratory endurance and muscle strength compared to men,^23^, ^40^ the present study shows that the fitness level of military troops may be maintained (or increased) even after accepting considerably more women into the military. This is an important finding, as reduced physical and occupational performance is frequently used as an argument against the inclusion of more women in the military and other physically demanding occupations.

## Supporting information


Supporting Information S1

Supporting Information S2

Supporting Information S3

## Data Availability

The data that support the findings of this study are available from the Norwegian Armed Forces HR and Conscription Centre, Hamar, Norway, but restrictions apply to the availability of these data, which were used under license for the current study, and so are not publicly available.
