# Supplementary material for: Temporal changes in physical fitness in Norwegian male and female military conscripts between 2006 and 2020
Source: Scand J Med Sci Sports. 2022 Nov 4;33(1):36–46. doi: 10.1111/sms.14238 (PMC10100210; doi:10.1111/sms.14238)
Supplement: Supplementary file 1 — Supporting Information S1 [file SMS-33-36-s002.docx]

SUPPORTING INFORMATION 1

**Equations used to predict maximal oxygen uptake (VO_2max_)**

3,000 meter run:

(1) Men: Ŷ = 17.5 + 2.57X

(2) Women: Ŷ = 14.6 + 2.48X

Ŷ = predicted VO_2max_ in mL∙kg^-1^∙min^-1^; X = 3,000 m average run speed in km·h^−1^.

Reference: Aandstad A. Estimation of maximal oxygen uptake from the 3,000 m run in adult men and women. J Sports Sci. 2021;39(15):1746-1753.

20 meter shuttle run test:

(1) Men: Ŷ = 2.75X + 28.8

(2) Women: Ŷ = 2.85X + 25.1

Ŷ = predicted VO_2max_ in mL∙kg^-1^∙min^-1^; X = last half-stage of the completed level

Reference: Stickland MK, Petersen SR, Bouffard M. Prediction of maximal aerobic power from the 20-m multi-stage shuttle run test. Can J Appl Physiol. 2003;28(2):272-282.
