# Supplementary material for: Temporal changes in physical fitness in Norwegian male and female military conscripts between 2006 and 2020
Source: Scand J Med Sci Sports. 2022 Nov 4;33(1):36–46. doi: 10.1111/sms.14238 (PMC10100210; doi:10.1111/sms.14238)
Supplement: Supplementary file 2 — Supporting Information S2 [file SMS-33-36-s003.docx]

SUPPORTING INFORMATION 2

**Description of data cleansing process (incl. exclusions and corrections)**

The percentages given in this document reflect the number of excluded/corrected data points divided by the total valid number of test results.

*3,000 meter test*

- Inclusion criterion was a test result between 07:01 and 59:59 min:sec. In total 233 results (0.25%) did not fulfill this criterion and were excluded (incl. 164 with result 00:00 min:sec).
- If a test result was reported as hours:min:sec (always with 00 sec), it was assumed that hours and minutes were mixed, and this was corrected accordingly. An example: 13:32:00 was changed to 13:32 min:sec. In total 397 results (0.42%) were corrected for this reason.
- For subjects with two reported 3,000 m test results, the last (newest) test result was excluded. In total 87 results (0.09%) were excluded for this reason.

*20 m shuttle run test*

- Inclusion criterion was a test result between 5 and 200 shuttles. In total 7 results (0.17%), all reporting 0 shuttles, did not fulfill this criterion and were excluded.

*Push-ups*

- Inclusion criterion was a test result ≤ 200 repetitions. In total 2 results (<0.01%) did not fulfill this criterion and were excluded.

*Sit-ups*

- Inclusion criterion was a test result ≤ 500 repetitions. In total 6 results (<0.01%) did not fulfill this criterion and were excluded.

*Pull-ups*

- Inclusion criterion was a test result ≤ 50 repetitions for vertical pull-ups, and ≤ 100 for horizontal pull-ups. In total 8 results (<0.01%) did not fulfill this criterion and were excluded.
- For subjects with two reported pull-up test results, the last (newest) test result was excluded. In total 106 results (0.11%) were excluded for this reason.

*Medicine ball throw*

- Inclusion criterion was a test result between 0.5 and 10.0 meter. In total 31 results (0.10%), all reporting 0.0 m, did not fulfill this criterion and were excluded.

*Standing long jump*

- Inclusion criterion was a test result between 0.5 and 3.70 meter. In total 53 results (0.17%) did not fulfill this criterion and were excluded.
