# Supplementary material for: Temporal changes in physical fitness in Norwegian male and female military conscripts between 2006 and 2020
Source: Scand J Med Sci Sports. 2022 Nov 4;33(1):36–46. doi: 10.1111/sms.14238 (PMC10100210; doi:10.1111/sms.14238)
Supplement: Supplementary file 3 — Supporting Information S3 [file SMS-33-36-s001.docx]

SUPPORTING INFORMATION 3

**Table 3S**. Change in 3,000 m run time in Norwegian recruits at entry to conscript service between 2006 and 2020. Data reported as mean min:sec ± SD, if not otherwise stated.

| Year | Men |  | Women |  | Both sexes |
| --- | --- | --- | --- | --- | --- |
| 2006 | 14:01 ± 02:26  (n = 6,400) |  | 16:46 ± 02:41  (n = 317) |  | 14:09 ± 02:31  (n = 6,717) |
| 2007 | 14:13 ± 02:41  (n = 5,229) |  | 16:52 ± 02:44  (n = 335) |  | 14:22 ± 02:45  (n = 5,564) |
| 2008 | 14:04 ± 02:28  (n = 6,222) |  | 16:43 ± 02:20  (n =514) |  | 14:16 ± 02:33  (n = 6,736) |
| 2009 | 13:55 ± 02:25  (n = 5,339) |  | 16:31 ± 02:34  (n = 550) |  | 14:09 ± 02:33  (n = 5,889) |
| 2010 | 13:56 ± 02:24  (n = 4,625) |  | 16:06 ± 02:13  (n = 441) |  | 14:08 ± 02:28  (n = 5,066) |
| 2011 | 14:01 ± 02:24  (n = 5,606) |  | 16:16 ± 02:15  (n = 597) |  | 14:14 ± 02:28  (n = 6,203) |
| 2012 | 14:02 ± 02:27  (n = 5,938) |  | 16:39 ± 02:23  (n = 640) |  | 14:17 ± 02:33  (n = 6,578) |
| 2013 | 13:58 ± 02:28  (n = 5,956) |  | 16:04 ± 02:36  (n = 843) |  | 14:14 ± 02:35  (n = 6,799) |
| 2014 | 13:47 ± 02:04  (n= 6,413) |  | 16:03 ± 02:14  (n = 1,328) |  | 14:11 ± 02:16  (n = 7,741) |
| 2015 | 13:51 ± 02:13  (n = 4,087) |  | 16:00 ± 02:16  (n = 846) |  | 14:13 ± 02:22  (n = 4,933) |
| 2016 | 13:25 ± 01:50  (n = 4,768) |  | 15:40 ± 02:01  (n = 1,384) |  | 13:55 ± 02:06  (n = 6,152) |
| 2017 | 13:29 ± 01:53  (n = 5,161) |  | 15:43 ± 02:07  (n = 1,793) |  | 14:04 ± 02:11  (n = 6,954) |
| 2018 | 13:28 ± 01:55  (n = 4,244) |  | 15:42 ± 02:03  (n = 1,803) |  | 14:08 ± 02:12  (n = 6,047) |
| 2019 | 13:12 ± 01:48  (n = 4,408) |  | 15:24 ± 01:56  (n = 2,175) |  | 13:56 ± 02:07  (n = 6,583) |
| 2020 | 13:09 ± 01:50  (n = 4,447) |  | 15:15 ± 01:56  (n = 2,414) |  | 13:54 ± 02:07  (n = 6,861) |
| All years | 13:48 ± 02:17  (n = 78,843) |  | 15:50 ± 02:13  (n = 15,980) |  | 14:08 ± 02:24  (n = 94,823) |
| *B* (95% CI) for linear trend (sec.) | -3.9 (-4.2, -3.7)  P < 0.001 |  | -6.8 (-7.3, -6.3)  P < 0.001 |  | -1.4 (-1.6, -1.2)  P < 0.001 |
| Mean (95% CI) change 2006 to 2020 (sec.) | -52 (-57, -47)  P < 0.001 |  | -90 (-105, -76)  P < 0.001 |  | -16 (-20, -11)  P < 0.001 |
| Mean change 2006 to 2020 (%) | -6.2 |  | -8.9 |  | -1.9 |
| Effect size (95% CI) for change 2006 to 2020 | 0.39 (0.36, 0.43) |  | 0.74 (0.62, 0.86) |  | 0.11 (0.08, 0.15) |

SUPPORTING INFORMATION 4

**Table 4S**. Change in 20 m shuttle run test performance in Norwegian recruits at entry to conscript service between 2017 and 2020. Data reported as mean number of shuttles ± SD, if not otherwise stated.

| Year | Men |  | Women |  | Both sexes |
| --- | --- | --- | --- | --- | --- |
| 2017 | 82.6 ± 20.5  (n = 508) |  | 56.9 ± 16.4  (n = 119) |  | 77.7 ± 22.2  (n = 627) |
| 2018 | 85.0 ± 19.7  (n = 874) |  | 62.0 ± 17.1  (n = 163) |  | 81.4 ± 21.0  (n = 1,037) |
| 2019 | 83.3 ± 19.8  (n = 1,089) |  | 61.1 ± 17.5  (n = 249) |  | 79.2 ± 21.2  (n = 1,338) |
| 2020 | 85.2 ± 19.8  (n = 936) |  | 64.9 ± 18.4  (n = 244) |  | 81.0 ± 21.2  (n = 1,180) |
| All years | 84.2 ± 19.9  (n = 3,407) |  | 61.9 ± 17.7  (n = 775) |  | 80.0 ± 21.3  (n = 4,182) |
| *B* (95% CI) for linear trend | 0.5 (-0.1, 1.2)  P = 0.123 |  | 2.2 (1.1, 3.4)  P < 0.001 |  | 0.6 (0.0, 1.2)  P = 0.062 |
| Mean (95% CI) change 2017 to 2020 | 2.6 (0.4, 4.8)  P = 0.019 |  | 8.1 (4.1, 12.0)  P < 0.001 |  | 3.3 (1.2, 5.4)  P = 0.002 |
| Mean change 2017 to 2020 (%) | 3.1 |  | 14.2 |  | 4.2 |
| Effect size (95% CI) for change 2017 to 2020 | 0.13 (0.02, 0.24) |  | 0.45 (0.23, 0.67) |  | 0.15 (0.06, 0.25) |

SUPPORTING INFORMATION 5

**Table 5S**. Change in estimated maximal oxygen uptake in Norwegian recruits at entry to conscript service between 2006 and 2020. Data reported as mean mL∙kg^-1^∙min^-1^ ± SD, if not otherwise stated.

| Year | Men |  | Women |  | Both sexes |
| --- | --- | --- | --- | --- | --- |
| 2006 | 51.3 ± 5.0  (n = 6,400) |  | 41.9 ± 3.9  (n = 317) |  | 50.9 ± 5.3  (n = 6,717) |
| 2007 | 51.0 ± 5.4  (n = 5,229) |  | 41.7 ± 4.0  (n = 335) |  | 50.5 ± 5.8  (n = 5,564) |
| 2008 | 51.2 ± 5.0  (n = 6,222) |  | 41.8 ± 3.5  (n = 514) |  | 50.5 ± 5.5  (n = 6,736) |
| 2009 | 51.6 ± 5.0  (n = 5,339) |  | 42.2 ± 3.9  (n = 550) |  | 50.7 ± 5.7  (n = 5,889) |
| 2010 | 51.5 ± 5.0  (n = 4,625) |  | 42.8 ± 3.6  (n = 441) |  | 50.8 ± 5.5  (n = 5,066) |
| 2011 | 51.3 ± 5.0  (n = 5,606) |  | 42.5 ± 3.6  (n = 597) |  | 50.5 ± 5.6  (n = 6,203) |
| 2012 | 51.3 ± 5.0  (n = 5,938) |  | 41.9 ± 3.6  (n = 640) |  | 50.4 ± 5.6  (n = 6,578) |
| 2013 | 51.5 ± 5.1  (n = 5,956) |  | 43.0 ± 4.1  (n = 843) |  | 50.4 ± 5.7  (n = 6,799) |
| 2014 | 51.7 ± 4.6  (n = 6,413) |  | 42.9 ± 3.6  (n = 1,328) |  | 50.2 ± 5.6  (n = 7,741) |
| 2015 | 51.7 ± 4.9  (n = 4,087) |  | 43.0 ± 3.7  (n = 846) |  | 50.2 ± 5.7  (n = 4,933) |
| 2016 | 52.6 ± 4.4  (n = 4,768) |  | 43.5 ± 3.5  (n = 1,384) |  | 50.6 ± 5.7  (n = 6,152) |
| 2017 | 52.5 ± 4.5  (n = 5,669) |  | 43.5 ± 3.7  (n = 1,912) |  | 50.2 ± 5.8  (n = 7,581) |
| 2018 | 52.7 ± 4.6  (n = 5,118) |  | 43.6 ± 3.7  (n = 1,966) |  | 50.1 ± 5.9  (n = 7,084) |
| 2019 | 53.1 ± 4.5  (n = 5,497) |  | 44.1 ± 3.7  (n = 2,424) |  | 50.4 ± 6.0  (n = 7,921) |
| 2020 | 53.3 ± 4.6  (n = 5,383) |  | 44.4 ± 3.7  (n = 2,658) |  | 50.4 ± 6.0  (n = 8,041) |
| All years | 51.9 ± 4.9  (n = 82,250) |  | 43.4 ± 3.8  (n = 16,755) |  | 50.4 ± 5.7  (n = 99,005) |
| *B* (95% CI) for linear trend | 0.15 (0.14, 0.15)  P < 0.001 |  | 0.19 (0.18, 0.21)  P < 0.001 |  | -0.03 (-0.04, -0.03)  P < 0.001 |
| Mean (95% CI) change 2006 to 2020 | 2.0 (1.8, 2.2)  P < 0.001 |  | 2.5 (2.1, 3.0)  P < 0.001 |  | -0.5 (-0.7, -0.3)  P < 0.001 |
| Mean change 2006 to 2020 (%) | 3.9 |  | 6.0 |  | -1.0 |
| Effect size (95% CI) for change 2006 to 2020 | 0.42 (0.38, 0.46) |  | 0.68 (0.56, 0.80) |  | 0.09 (0.05, 0.12) |

SUPPORTING INFORMATION 6

**Table 6S**. Change in push-up performance in Norwegian recruits at entry to conscript service between 2006 and 2016. Data reported as mean number of repetitions ± SD, if not otherwise stated.

| Year | Men |  | Women |  | Both sexes |
| --- | --- | --- | --- | --- | --- |
| 2006 | 25.3 ± 12.6  (n = 6,650) |  | 10.4 ± 8.4  (n = 340) |  | 24.6 ± 12.8  (n = 6,990) |
| 2007 | 23.8 ± 12.4  (n = 5,587) |  | 8.8 ± 8.3  (n = 367) |  | 22.9 ± 12.7  (n = 5,954) |
| 2008 | 26.1 ± 12.8  (n = 6,116) |  | 9.9 ± 8.4  (n = 523) |  | 24.8 ± 13.3  (n = 6,639) |
| 2009 | 26.9 ± 13.0  (n = 5,713) |  | 10.1 ± 9.4  (n = 577) |  | 25.3 ± 13.6  (n = 6,290) |
| 2010 | 26.8 ± 13.5  (n = 4,821) |  | 11.0 ± 10.1  (n = 464) |  | 25.5 ± 14.0  (n = 5,285) |
| 2011 | 27.6 ± 13.3  (n = 5,744) |  | 11.2 ± 9.3  (n = 587) |  | 26.1 ± 13.8  (n = 6,331) |
| 2012 | 27.0 ± 13.3  (n = 5,773) |  | 9.7 ± 8.6  (n = 645) |  | 25.3 ± 13.9  (n = 6,418) |
| 2013 | 26.5 ± 12.7  (n = 5,954) |  | 10.5 ± 9.5  (n = 854) |  | 24.5 ± 13.4  (n = 6,808) |
| 2014 | 26.3 ± 12.3  (n = 6,668) |  | 10.0 ± 8.4  (n = 1,361) |  | 23.5 ± 13.2  (n = 8,029) |
| 2015 | 26.1 ± 11.6  (n = 4,082) |  | 9.7 ± 8.4  (n = 851) |  | 23.3 ± 12.7  (n = 4,933) |
| 2016 | 27.0 ± 11.7  (n = 5,020) |  | 10.6 ± 8.7  (n = 1,455) |  | 23.3 ± 13.1  (n = 6,475) |
| All years | 26.3 ± 12.7  (n = 62,128) |  | 10.2 ± 8.9  (n = 8,024) |  | 24.5 ± 13.4  (n = 70,152) |
| *B* (95% CI) for linear trend | 0.17 (0.14, 0.20)  P < 0.001 |  | 0.03 (-0.04, 0.09)  P = 0.449 |  | -0.10 (-0.13, -0.07)  P < 0.001 |
| Mean (95% CI) change 2006 to 2016 | 1.7 (1.2, 2.1)  P < 0.001 |  | 0.1 (-0.9, 1.2)  P = 0.790 |  | -1.3 (-1.7, -0.9)  P < 0.001 |
| Mean change 2006 to 2016 (%) | 6.7 |  | 1.0 |  | -5.3 |
| Effect size (95% CI) for change 2006 to 2016 | 0.14 (0.10, 0.17) |  | 0.02 (-0.10, 0.13) |  | 0.10 (0.07, 0.13) |

SUPPORTING INFORMATION 7

**Table 7S**. Change in sit-up performance in Norwegian recruits at entry to conscript service between 2006 and 2016. Data reported as mean number of repetitions ± SD, if not otherwise stated.

| Year | Men |  | Women |  | Both sexes |
| --- | --- | --- | --- | --- | --- |
| 2006 | 31.2 ± 19.6  (n = 6,572) |  | 26.5 ± 22.3  (n = 345) |  | 31.0 ± 19.8  (n = 6,917) |
| 2007 | 28.8 ± 18.6  (n = 5,605) |  | 23.4 ± 13.6  (n = 364) |  | 28.5 ± 18.4  (n = 5,969) |
| 2008 | 31.2 ± 18.6  (n = 6,042) |  | 24.9 ± 15.2  (n = 525) |  | 30.7 ± 18.4  (n = 6,567) |
| 2009 | 31.2 ± 18.5  (n = 5,624) |  | 23.9 ± 17.2  (n = 565) |  | 30.5 ± 18.5  (n = 6,189) |
| 2010 | 30.9 ± 19.5  (n = 4,771) |  | 26.0 ± 17.1  (n = 455) |  | 30.5 ± 19.3  (n = 5,226) |
| 2011 | 33.2 ± 22.5  (n = 5,731) |  | 28.2 ± 22.6  (n = 603) |  | 32.7 ± 22.6  (n = 6,334) |
| 2012 | 31.2 ± 16.8  (n = 5,681) |  | 25.2 ± 16.8  (n = 642) |  | 30.6 ± 16.9  (n = 6,323) |
| 2013 | 30.1 ± 14.8  (n = 5,939) |  | 26.7 ± 16.3  (n = 851) |  | 29.6 ± 15.0  (n = 6,790) |
| 2014 | 31.8 ± 16.3  (n = 6,618) |  | 26.7 ± 17.4  (n = 1,361) |  | 30.9 ± 16.6  (n = 7,979) |
| 2015 | 34.4 ± 16.7  (n = 4,097) |  | 29.8 ± 15.1  (n = 853) |  | 33.6 ± 16.5  (n = 4,950) |
| 2016 | 34.7 ± 17.5  (n = 5,020) |  | 31.2 ± 16.1  (n = 1,466) |  | 33.9 ± 17.2  (n = 6,486) |
| All years | 31.6 ± 18.3  (n = 61,700) |  | 27.3 ± 17.3  (n = 8,030) |  | 31.1 ± 18.3  (n = 69,730) |
| *B* (95% CI) for linear trend | 0.32 (0.28, 0.37)  P < 0.001 |  | 0.64 (0.52, 0.76)  P < 0.001 |  | 0.28 (0.24, 0.32)  P < 0.001 |
| Mean (95% CI) change 2006 to 2016 | 3.5 (2.8, 4.2)  P < 0.001 |  | 4.8 (2.7, 6.8)  P < 0.001 |  | 2.9 (2.3, 3.6)  P < 0.001 |
| Mean change 2006 to 2016 (%) | 11.2 |  | 18.1 |  | 9.4 |
| Effect size (95% CI) for change 2006 to 2016 | 0.19 (0.15, 0.22) |  | 0.27 (0.16, 0.39) |  | 0.16 (0.12, 0.19) |

SUPPORTING INFORMATION 8

**Table 8S**. Change in pull-up (vertical) performance in Norwegian recruits at entry to conscript service between 2006 (2017) and 2020. Data reported as mean number of repetitions ± SD, if not otherwise stated.

| Year | Men |  | Women |  | Both sexes |
| --- | --- | --- | --- | --- | --- |
| 2006 | 4.4 ± 3.9  (n = 6,579) |  | N/A |  | N/A |
| 2007 | 4.3 ± 3.9  (n = 5,641) |  | N/A |  | N/A |
| 2008 | 5.2 ± 5.0  (n = 5,918) |  | N/A |  | N/A |
| 2009 | 5.5 ± 5.0  (n = 5,654) |  | N/A |  | N/A |
| 2010 | 5.2 ± 4.4  (n = 4,651) |  | N/A |  | N/A |
| 2011 | 5.4 ± 4.5  (n = 5,725) |  | N/A |  | N/A |
| 2012 | 5.2 ± 4.5  (n = 5,739) |  | N/A |  | N/A |
| 2013 | 5.6 ± 4.6  (n = 5,996) |  | N/A |  | N/A |
| 2014 | 5.8 ± 4.7  (n = 6,678) |  | N/A |  | N/A |
| 2015 | 6.1 ± 4.7  (n = 4,077) |  | N/A |  | N/A |
| 2016 | 6.4 ± 4.8  (n = 4,981) |  | N/A |  | N/A |
| 2017 | 6.6 ± 4.6  (n = 5,672) |  | 0.7 ± 1.7  (n = 1,869) |  | 5.1 ± 4.8  (n = 7,541) |
| 2018 | 6.8 ± 4.6  (n = 5,135) |  | 0.8 ± 1.9  (n = 1,941) |  | 5.1 ± 4.8  (n = 7,076) |
| 2019 | 7.4 ± 4.9  (n = 5,547) |  | 0.8 ± 2.0  (n = 2,435) |  | 5.4 ± 5.2  (n = 7,982) |
| 2020 | 8.0 ± 5.1  (n = 5,396) |  | 1.0 ± 2.3  (n = 2,671) |  | 5.7 ± 5.5  (n = 8,067) |
| All years | 5.8 ± 4.7  (n = 83,389) |  | 0.8 ± 2.0  (n = 8,916) |  | 5.3 ± 5.1  (n = 30,666) |
| *B* (95% CI) for linear trend | 0.22 (0.21, 0.23)  P < 0.001 |  | 0.10 (0.07, 0.14)  P < 0.001 |  | 0.21 (0.16, 0.26)  P < 0.001 |
| Mean (95% CI) change 2006 (2017) to 2020 | 3.7 (3.5, 3.8)  P < 0.001 |  | 0.3 (0.2, 0.5)  P < 0.001 |  | 0.6 (0.4, 0.8)  P < 0.001 |
| Mean change 2006 (2017) to 2020 (%) | 84.1 |  | 42.9 |  | 11.8 |
| Effect size (95% CI) for change 2006 (2017)  to 2020 | 0.82 (0.78, 0.85) |  | 0.16 (0.10, 0.22) |  | 0.12 (0.09, 0.15) |

N/A, not available

SUPPORTING INFORMATION 9

**Table 9S**. Change in pull-up (horizontal) performance in Norwegian female recruits at entry to conscript service between 2006 and 2016. Data reported as mean number of repetitions ± SD, if not otherwise stated.

| Year | Women |  |
| --- | --- | --- |
| 2006 | 6.5 ± 5.1  (n = 345) |  |
| 2007 | 5.6 ± 4.7  (n = 358) |  |
| 2008 | 6.4 ± 5.0  (n = 496) |  |
| 2009 | 5.6 ± 4.8  (n = 574) |  |
| 2010 | 6.1 ± 5.3  (n = 454) |  |
| 2011 | 6.4 ± 5.5  (n = 593) |  |
| 2012 | 6.2 ± 5.3  (n = 647) |  |
| 2013 | 6.6 ± 5.6  (n = 866) |  |
| 2014 | 7.1 ± 6.1  (n = 1,363) |  |
| 2015 | 5.5 ± 5.6  (n = 855) |  |
| 2016 | 5.8 ± 5.7  (n = 1,428) |  |
| All years | 6.2 ± 5.5  (n = 7,979) |  |
| *B* (95% CI) for linear trend | 0.00 (-0.04, 0.04)  P = 0.853 |  |
| Mean (95% CI) change 2006 to 2016 | -0.7 (-1.3, 0.0)  P = 0.040 |  |
| Mean change 2006 to 2016 (%) | -10.8 |  |
| Effect size (95% CI) for change 2006 to 2016 | 0.12 (0.01, 0.24) |  |

SUPPORTING INFORMATION 10

**Table 10S**. Change in medicine ball throw performance in Norwegian recruits at entry to conscript service between 2017 and 2020. Data reported as mean centimeter ± SD, if not otherwise stated.

| Year | Men |  | Women |  | Both sexes |
| --- | --- | --- | --- | --- | --- |
| 2017 | 453 ± 53  (n =5,809) |  | 320 ± 42  (n = 1,990) |  | 419 ± 77  (n = 7,799) |
| 2018 | 453 ± 54  (n = 5,158) |  | 320 ± 39  (n = 1,999) |  | 416 ± 78  (n = 7,157) |
| 2019 | 449 ± 54  (n = 5,632) |  | 313 ± 39  (n = 2,500) |  | 407 ± 80  (n = 8,132) |
| 2020 | 450 ± 55  (n = 5,445) |  | 319 ± 42  (n = 2,702) |  | 406 ± 80  (n = 8,147) |
| All years | 451 ± 54  (n = 22,044) |  | 318 ± 41  (n = 9,191) |  | 412 ± 79  (n = 31,235) |
| *B* (95% CI) for linear trend | -1.4 (-2.1, -0.8)  P < 0.001 |  | -0.8 (-1.5, 0.0)  P = 0.041 |  | -4.7 (-5.4, -3.9)  P < 0.001 |
| Mean (95% CI) change 2017 to 2020 | -3 (-5, -1)  P < 0.001 |  | -1 (-3, 1)  P = 0.411 |  | -13 (-15, -10)  P < 0.001 |
| Mean change 2017 to 2020 (%) | -0.7 |  | -0.3 |  | -3.1 |
| Effect size (95% CI) for change 2017 to 2020 | 0.06 (0.03, 0.10) |  | 0.02 (-0.03, 0.08) |  | 0.16 (0.13, 0.20) |

SUPPORTING INFORMATION 11

**Table 11S**. Change in standing long jump performance in Norwegian recruits at entry to conscript service between 2017 and 2020. Data reported as mean centimeter ± SD, if not otherwise stated.

| Year | Men |  | Women |  | Both sexes |
| --- | --- | --- | --- | --- | --- |
| 2017 | 227 ± 22  (n = 5,766) |  | 184 ± 22  (n = 1,988) |  | 216 ± 29  (n = 7,754) |
| 2018 | 228 ± 22  (n = 5,154) |  | 185 ± 21  (n = 1,999) |  | 216 ± 29  (n = 7,153) |
| 2019 | 229 ± 22  (n = 5,622) |  | 185 ± 20  (n = 2,486) |  | 216 ± 29  (n = 8,108) |
| 2020 | 231 ± 21  (n = 5,421) |  | 188 ± 19  (n = 2,697) |  | 217 ± 29  (n = 8,118) |
| All years | 229 ± 22  (n = 21,963) |  | 186 ± 20  (n = 9,170) |  | 216 ± 29  (n = 31,133) |
| *B* (95% CI) for linear trend | 1.3 (1.0, 1.5)  P < 0.001 |  | 1.4 (1.1, 1.8)  P < 0.001 |  | 0.2 (-0.1, 0.5)  P = 0.182 |
| Mean (95% CI) change 2017 to 2020 | 4 (3, 5)  P < 0.001 |  | 4 (3, 6)  P < 0.001 |  | 1 (0, 2)  P = 0.086 |
| Mean change 2017 to 2020 (%) | 1.8 |  | 2.2 |  | 0.5 |
| Effect size (95% CI) for change 2017 to 2020 | 0.18 (0.15, 0.22) |  | 0.22 (0.16, 0.28) |  | 0.03 (0.00, 0.06) |
